# Removal of the Pulp by the Use of Cocaine

**Published:** 1891-05

**Authors:** Edward C. Briggs


### REMOVAL OF THE PULP BY THE USE OF COCAINE.[1]

BY EDWARD C. BRIGGS, M.D., D.M.D.

THE operation I am about to describe I have never seen mentioned; as far as I know, it is original. At all events, to any who have not discovered it it will come as a blessing, for it does away with one of the most troublesome conditions with which we are called upon to deal.

Briefly and to the point, it is the removal of the pulp by the use of cocaine. The method and detail of the operation I can best bring out by describing the cases upon which I have operated.

CASE I.—Mrs. M. came to me in May with an aching pulp in the left superior second bicuspid. I found an exposure, and dressed it to reduce the inflammation. After a few days, the tooth being comfortable, I applied arsenious acid to destroy the pulp. It acted in the not unusual way,—that is, it caused a violent inflammation.

When I saw the patient May 17, 1890, she was suffering terribly. After I had applied local anæsthetics without producing any effect, I partly anæsthetized the patient, to give her, at least, temporary relief, and took the opportunity to apply more closely and exactly to the pulp an anæsthetic. When the patient came out of the anæsthesia, the tooth began to ache again with redoubled vigor. It was then that it occurred to me to try to *inject* my solution of cocaine into the pulp. I had, of course, previously applied it very freely. I therefore took my hypodermic syringe and filled it with a twenty-per-cent. solution of cocaine, and, pressing the

---

[1] Read before the Harvard Odontological Society, Boston, Mass., October 23, 1890.

point firmly against the opening into the pulp, slowly injected it. At the first pressure there was a slight start; but, waiting a few seconds, I was able to force the quantity in the syringe against the pulp with considerable pressure, the excess, of course, escaping around the point. This force I consider a great factor, for I do not think the cocaine goes into the pulp, but is carried by the pressure of the piston up around that organ. In the case I am describing, the pain ceased immediately.

It occurred to me that perhaps there was more than cessation of pain, that perhaps I could now remove the pulp. This was found to be the case, and it was removed without the patient feeling any pain.

I immediately communicated this fact to Dr. Hamilton and Dr. Charles Briggs, urging them to give it a trial. I hope to have them relate their experiences to you to-night.

Case II.—A. M. had been an invalid for two years, in such a state of health as to prohibit any dental attendance. When I saw him in March, he had an aching right superior first molar. After removing the decayed and softened dentine, except a thin layer over the pulp, I dressed the tooth and dismissed him. He came again, by appointment, May 28. He then informed me that the tooth was more or less troublesome all the time. Feeling that it was useless to temporize with it, I removed the dressing, and with a slight movement, with spoon excavator, exposed the pulp. I could just get the point of the syringe through the opening. Before injecting, I applied on cotton some of the cocaine, so that putting the syringe-point in did not occasion pain.

Here, again, I began slowly to inject, and, after the first slight start, there was no more feeling while injecting. In the last half of the amount in the syringe I pressed the piston down rapidly.

The cavity being distal, and somewhat inaccessible, I took my engine, and with this instrument went down through the crown and removed the entire pulp without further sensation. Not having the time, I did not fill the roots, but gave the patient another appointment. This I found to be a mistake, for when the patient came again, I had great difficulty in getting into the roots. Although I had seen the entire pulp on the broach, the root-canals were as sensitive as though the pulp was still there. This I have found to be true in other cases. I now, therefore, fill the roots before the benumbing effect of the cocaine upon the nerve-fibrils has passed off.

The very fact that these are not destroyed, as is the case with arsenious acid and other caustics, is greatly in favor of this method. The tooth in this case is not at all *dead*, it is only pulpless.

CASE III.—In this case I was ready to put on a gold crown over a much broken-down third molar, when I found the pulp ought to be exposed; accordingly I opened into it and injected the cocaine, removed the pulp, filled the roots, and put on the crown, with no other pain, then or since, except a slight sensation when the pulp first felt the impact of the cocaine.

CASE IV.—This patient had a fall, striking on the front teeth. The left superior central incisor was broken off, exposing the pulp. This was destroyed with arsenious acid before I had begun the use of cocaine. In June last I discovered a little discoloration of the right central, and, drilling into the pulp-chamber, obtained a drop of pus, but found the pulp beyond alive. Treated it the same as former cases, and there was little or no pain.

CASE V.—This was one of those pulps which, having been capped, behave well for a year or so and then begin to die. I opened into the pulp-chamber, washed out the decomposed portion, fitted the point of syringe, wrapped with cotton tightly in the opening, and injected as before described. The pulp was removed with a barbed broach and the root filled.

CASE VI.—This was a case that had been under treatment for some time. There was life in a portion of each root, which resisted the action of arsenious acid. The cocaine was forced into the root, causing no more pain than would be felt by the exploring probe. The troublesome remnant was removed, and the canals filled without further pain.

CASE VII.—This was a superior third molar, which had ached badly before patient came to me. Found an exposure, forced the cocaine in, removed the pulp, and filled the root, without the patient fully grasping the fact that I was doing more than the ordinary excavating for simple cavity.

CASE VIII.—Case similar to Case 4. Young man with delicate teeth. Right superior central incisor had small gold filling in mesial, put in before I saw him, and small gutta-percha in distal. The patient's mother discovered discoloration, and sent him in to see me. I drilled and got pus, but found pulp in root alive. Injected cocaine, removed the pulp, and filled root, causing only a little pain. Patient said he "did not mind it."

CASE IX.—History of an old exposure successfully capped for some years. Gold-filling had broken out, and caries had again endangered pulp.

After removing the pulp, by means of cocaine, and filling root, patient expressed himself as having had the easiest time he had ever experienced ; which simply indicates that the treatment could not have been very painful.

Case X.—This patient lives in New York. Once a year I had dressed an old exposure in left superior first molar, which had resisted arsenious acid in former times. This pulp I disposed of in the morning, and filled the roots. In the afternoon put in a large mesial-crown gold filling; no trouble then or since.

Case XI.—This patient has delicate teeth. An ordinary summer absence will develop a cavity reaching the pulp.

This time it was a buccal cavity in left superior second molar. This could have been capped, but, knowing the irritability of the pulps and the danger of new decay undermining the dressing and undoing my treatment, I decided to remove the pulp.

By careful cutting with spoon excavator I obtained a small opening to the pulp with no more pain than might occur in ordinary excavating. Got the point of the syringe firmly fixed in the opening, held it steady with both hands, and then asked the patient to press the piston down and to stop when it hurt him. He stopped once when he had pressed down about a sixteenth of an inch, but started on immediately, evidently not considering himself much hurt, and pressed clear down. I opened the pulp-chamber from the crown, removed the pulp without causing any pain, and filled the roots.

There is one point to be mentioned which has not been brought out in cases cited, and that is the excessive bleeding after the injection of the cocaine.

This is sometimes a matter of considerable annoyance, delaying the filling of the roots.

